# Successful Protein Extraction from Over-Fixed and Long-Term Stored Formalin-Fixed Tissues

**DOI:** 10.1371/journal.pone.0016353

**Published:** 2011-01-31

**Authors:** Claudia Wolff, Christina Schott, Peter Porschewski, Bilge Reischauer, Karl-Friedrich Becker

**Affiliations:** 1 Institute of Pathology, Technische Universität München, Munich, Germany; 2 Qiagen GmbH, Hilden, Germany; Agency for Science, Technology and Research (A*STAR), Singapore

## Abstract

One of the major breakthroughs in molecular pathology during the last decade was the successful extraction of full-length proteins from formalin-fixed and paraffin-embedded (FFPE) clinical tissues. However, only limited data are available for the protein extraction efficiency of over-fixed tissues and FFPE blocks that had been stored for more than 15 years in pathology archives. In this study we evaluated the protein extraction efficiency of FFPE tissues which had been formalin-fixed for up to 144 hours and tissue blocks that were stored for 20 years, comparing an established and a new commercial buffer system. Although there is a decrease in protein yield with increasing fixation time, the new buffer system allows a protein recovery of 66% from 144 hours fixed tissues compared to tissues that were fixed for 6 hours. Using the established extraction procedure, less than 50% protein recovery was seen. Similarly, the protein extraction efficiency decreases with longer storage times of the paraffin blocks. Comparing the two buffer systems, we found that 50% more proteins can be extracted from FFPE blocks that were stored for 20 years when the new buffer system is used. Taken together, our data show that the new buffer system is superior compared to the established one. Because tissue fixation times vary in the routine clinical setting and pathology archives contain billions of FFPE tissues blocks, our data are highly relevant for research, diagnosis, and treatment of disease.

## Introduction

The use of formalin as a fixative has been standard in the clinical routine for more than 100 years and still is. For quite a long time it seemed impossible to use formalin-fixed, paraffin-embedded (FFPE) tissues for quantitative proteome analysis [1]–[3]. However in the last few years several groups - including our own - described successful protein extraction from FFPE tissues [4]–[12]. It could be demonstrated that the extracted proteins are non-degraded, full-length, and immunoreactive and for this reason suitable for standard methods as western blot, protein microarray [5] and 2D gel electrophoresis [4]. This is a great advantage for research as with this technique it is not required to use rare fresh frozen material but one can resort to the large FFPE tissue archives of most hospitals worldwide. But it is of great importance that this method is not only used for research purposes but will be integrated in clinical routine, too, especially as individualised therapies gain more and more impact for patient diagnosis and therapy decision. However to reach this goal several aspects of protein extraction from FFPE tissue have to be considered. For use in routine diagnostic a successful extraction protocol should be fast, effective, standardized, and reliable. Another important issue that shouldn't be disregarded is the dissimilar pre-analytical treatment of different tissue samples. Due to practical and organisational reasons fixation times in clinical routine may vary from tissue sample to tissue sample. The minimal fixation time, depending on the tissue size, should be around 6 h, but samples may also stay in formalin for several days, e.g. if they arrive shortly before the weekend or public holidays. Especially those extendedly fixed tissues are a hurdle for efficient protein extraction from FFPE tissue. In this manuscript we addressed some of these issues and found that a new buffer system is superior compared to an established system when proteins are extracted from over-fixed or long-term stored tissues.

## Results

### Protein yields in various tissue types

**Figure 1:**
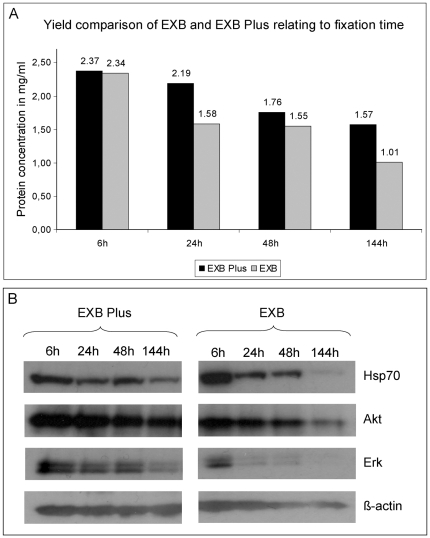
Comparison of proteins extracted from five different tissues with EXB and EXB Plus, respectively. **A** Using EXB Plus considerably higher lysate concentrations could be obtained for each of the tissue types. **B** Western blot analysis of E-Cadherin, Erk and β-actin in five different tissues. For both buffers we obtained clear bands in the western blot, confirming that we were able to extract non-degraded, full-length, and immunoreactive proteins. In addition it is distinguishable that the extracted protein amount from all five tissues of all three analysed proteins is higher using EXB Plus.

We used five different tissue types (Barrett's carcinoma, pancreas carcinoma, non-tumourous colon, gastric cancer and a lymph node sample) to test the protein yields that can be obtained from routinely processed FFPE tissues comparing two commercial buffer systems, EXB and EXB Plus (**Figure 1A**). For both approaches we extracted proteins from five 10 µm sections with an approximate area of about 0.5 cm^2^ of a formalin-fixed tissue sample in 100 µl of extraction buffer. Total protein concentrations of the extracts ranged from 1.66 mg/ml to 5.33 mg/ml using the EXB extraction method. With EXB Plus we obtained yields ranging from 2.58 mg/ml to 8.62 mg/ml. It could be shown that in all applied tissues the protein amount in extracts obtained using EXB Plus was about two-times higher compared to samples from EXB based extractions.

In a next step, we performed western blot analysis to ensure reliable extraction of non-degraded, full-length, and immunoreactive proteins comparing EXB Plus and EXB extraction buffers. In **Figure 1B** the results of a western blot analysis of Erk, β-actin and the membrane protein E-Cadherin are shown. For this purpose the tenth part of each extraction was used. This gave us the possibility to not only compare the total protein extraction yield but also the amount of individual proteins. It is distinguishable that with EXB Plus it is possible to extract higher amounts from all five tissues and of all three analysed proteins compared to EXB. Furthermore as we obtained clear bands at the right molecular weight for both buffers, we could confirm the integrity of the extracted proteins. Independent of the buffer no E-Cadherin expression could be detected for pancreas carcinoma and the lymph node sample. This is not astonishing as lymphocytes do not express the protein and down-regulation of E-Cadherin in pancreas carcinoma may occur [13], [14].

### Comparison of protein yields from tissue samples from different hospitals

**Figure 2:**
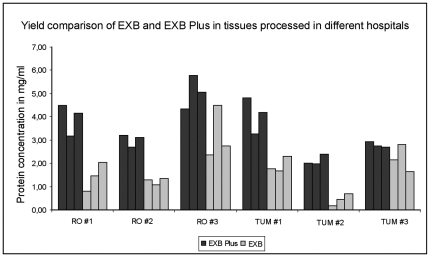
Protein amounts gained by extraction from FFPE tissues from two different hospitals in three technical replicates. EXB Plus allowed high protein yields independently of the tumor sample or the hospital the tissue was processed in. Additionally only minimal variations between the replicates (mean standard deviation: 0.47 mg/ml) could be detected.

To show that this advantage of EXB Plus is not only true for tissues processed in our institute we compared the extraction yield from two different hospitals (Klinikum rechts der Isar, Munich and Klinikum Rosenheim, Rosenheim). For this purpose proteins were extracted from three different tissue samples from both hospitals, in three technical replicates using both buffers. This allowed us to determine the variation between different extractions. As **Figure 2** shows EXB Plus resulted in high protein yields from both hospitals and in only minimal variations between the replicates (mean standard deviation: 0.47 mg/ml).

### Protein extraction from FFPE tissues from xerograph mouse models

**Figure 3:**
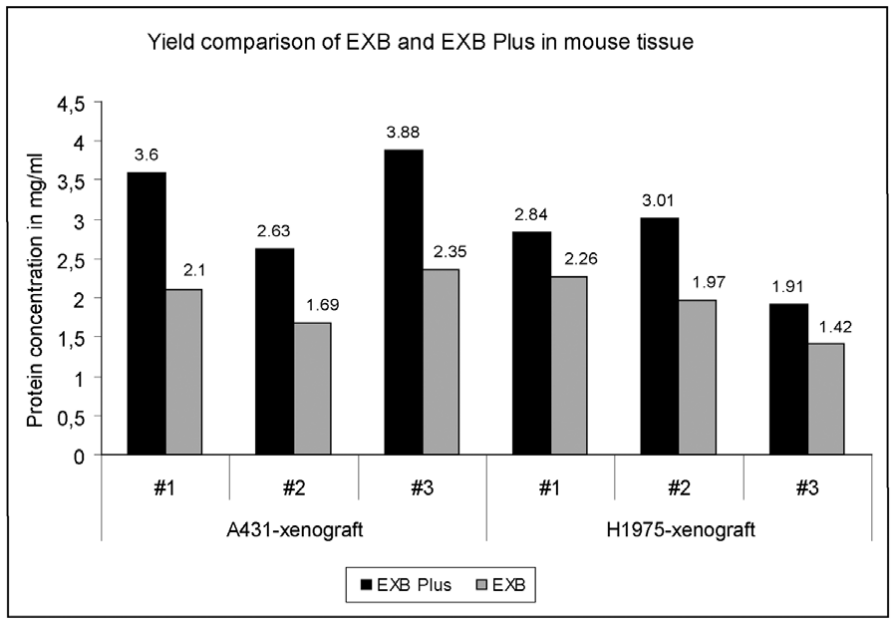
Protein extraction from FFPE samples from two different xenograft mouse models. For both mouse models EXB Plus resulted in higher protein yields compared to EXB. This shows the advantage of EXB Plus independently of the organism.

To determine the applicability of EXB Plus not only in human but also in other organisms, we used tumors from two different xenograft-mouse models (A431- and H1975-xenograft) and extracted proteins from three different mice for each mouse model (**Figure 3**). It could be shown that in all six samples the protein amount in extracts obtained using EXB Plus were higher than those gained with EXB. Similar results were achieved with extractions from rat tissue (data not shown). These findings reveal that EXB Plus can be used for efficient protein extraction from FFPE tissue samples independently of its origin.

### Protein extraction from FFPE tissues versus fresh frozen tissues

**Figure 4:**
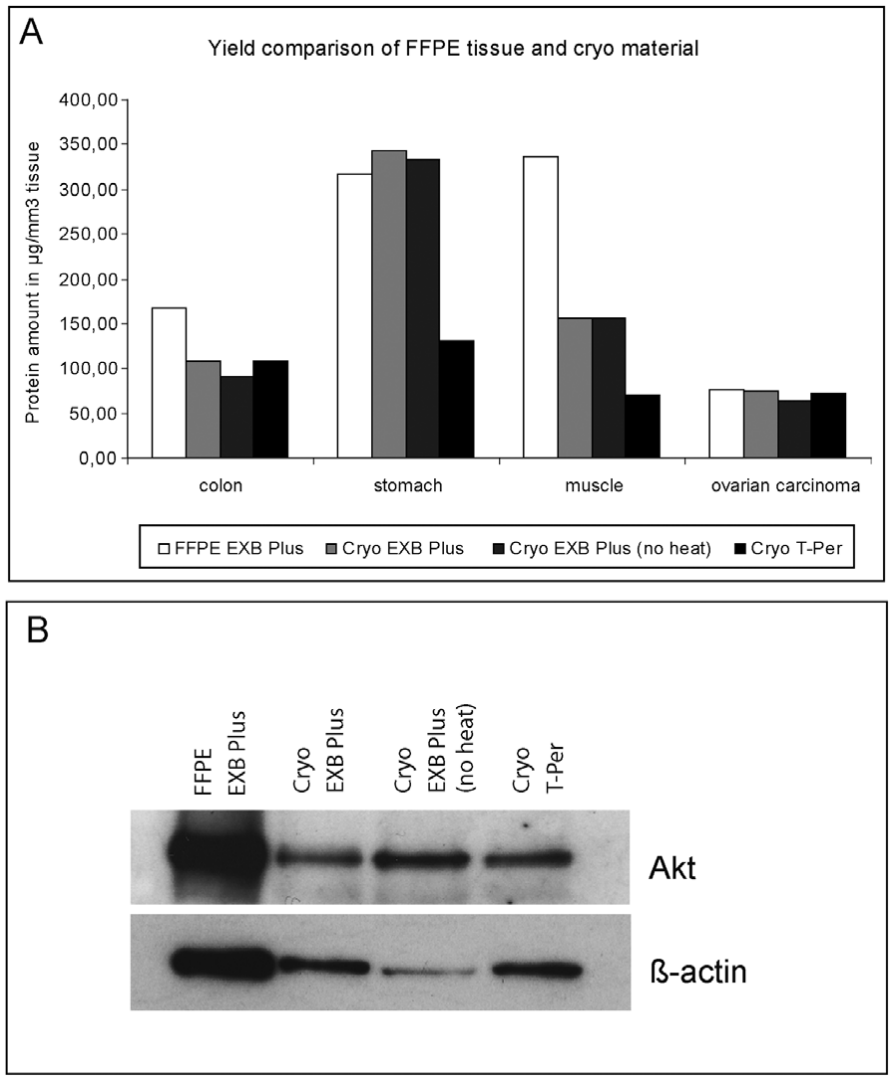
Comparison of proteins extracted from four FFPE tissues and the corresponding cryo tissues with EXB Plus. **A** Cryo tissue was additionally extracted with EXB Plus but without any heat and T-Per, a buffer designed for extraction of cryo material. To be able to compare protein yields from different sized tissue pieces the protein amount is calculated per mm^3^ of used tissue. The yields extracted from cryo material with EXB Plus or T-Per were equal, or especially for stomach tissue even higher in the EXB Plus extracts. Cryo tissue compared to FFPE material discloses a loss of protein amount in two tissues (colon and muscle) and no difference in the two others (ovarian carcinoma and stomach). **B** Western blot analysis of protein lysates from colon FFPE and cryo material. For both tissue types we obtained clear bands in the western blot, confirming that we were able to extract non-degraded, full-length, and immunoreactive proteins. In addition it is visible that the extracted protein amount from FFPE is higher than from fresh-frozen tissue, but hardly any difference could be seen for cryo material extracted using EXB Plus compared to a standard buffer for cryo extraction (T-Per).

To compare protein extraction efficiencies between FFPE material and fresh frozen tissues, proteins should be extracted in the same buffer system, as the protein composition may be influenced by the extraction buffer. For this reason we extracted proteins from four FFPE tissues and the corresponding cryo tissues with EXB Plus. Cryo material was additionally extracted using EXB Plus but without the two heating steps as these are usually not necessary for fresh frozen tissue. As reference we extracted cryo tissue with the commercial buffer T-Per (Thermo-Fisher, Rockford, USA), a dedicated cryo extraction buffer, according to the manufacturer's instructions. The obtained results are shown in **Figure 4A**. No difference could be observed for the extraction with EXB Plus from fresh frozen material with or without heat whereas for extraction from FFPE tissue heat (20 min at 100°C; 2 h at 80°C) is implicitly necessary (data not shown). For two tissues (ovarian carcinoma, stomach) we gained equal protein amounts from cryo material and FFPE tissue. For the two others (colon, muscle) the protein amount obtained from cryo material was lower. However, if these amounts from cryo tissue are compared to the amounts extracted with T-Per, it appears that they are still as high as the ones extracted from T-Per – or even higher (**Figure 4A**). Most importantly as **Figure 4B** shows we obtained clear bands at the right molecular weight for FFPE and fresh frozen tissue. This confirms the integrity of the extracted proteins from both materials. As the tenth part of each extraction was used in this western blot, the relation of the extracted protein amounts (according to the different buffers and tissues) as described above could be made visible.

All together our data indicate that even though EXB Plus is designed for extraction from FFPE material, it could also be used for fresh frozen tissues, if required.

### Protein yields from over-fixed tissues

**Figure 5:**
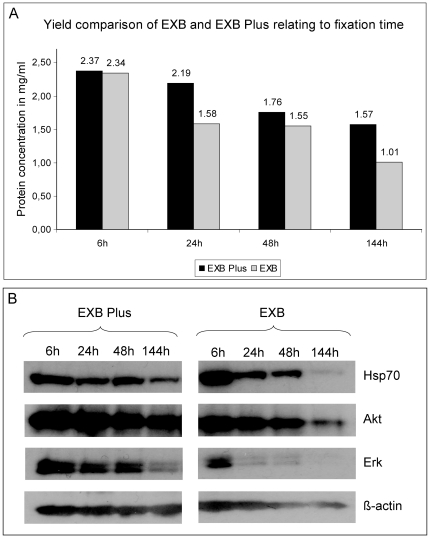
Protein amounts and western blot analysis of lysates extracted from lymph node samples fixed for 6 h, 24 h, 48 h and 144 h, respectively. For a short fixation time no real difference could be detected between the two buffers tested. But already at the standard fixation time of 24 h EXB Plus shows 25% higher protein yield. For a fixation time of 144 h the difference increased to 30% higher protein yield compared to EXB. **B** Western blot analysis of Hsp70, Akt, Erk and β-actin in lymph node samples fixed for 6 h, 24 h, 48 h or 144 h. Protein yield decreases with extension of fixation for EXB, for all four analysed proteins. In contrast, using EXB Plus the decline is much less pronounced. All eight samples detected with one antibody were run on one gel and detected under exactly the same conditions (e.g. blocking, washing steps, exposure time).

It is well known that, due to practical and organisational reasons, in the clinical routine the fixation time of tissues varies. The minimal fixation time, depending on the tissue size, should be around 6 h but if samples arrive shortly before the weekend or public holidays they could also stay in formalin for several days. Taking this into account, we extracted proteins (with EXB and EXB Plus) from lymph node samples that were fixed for 6 h, 24 h, 48 h, or 144 h (**Figure 5A**). For the shortest fixation time (6 h), no real difference could be detected between the two buffers tested. But already at the standard fixation time of 24 h extraction with EXB Plus led to an explicitly higher protein yield. The elevated protein yield remained for fixation times of 48 h and 144 h using EXB Plus. Although lymph nodes were the tissue with the lowest obtained protein amounts of all tissues analysed, we were still able to extract a suitable amount of protein (1.57 mg/ml in 100 µl) even from the 144 h fixed sample using EXB Plus (**Figure 5A**).

As next step, we investigated how proteins from these differently fixed samples behave in western blot analysis. For this purpose we analysed the lysates extracted with EXB and EXB Plus for four proteins: The heat shock protein Hsp70, the protein kinase B (Akt), the extracellular signal-regulated kinase (Erk) and β-actin (**Figure 5B**). The results clearly show that even highly abundant proteins, like Hsp70, are only poorly detectable using EXB, if the sample was fixed extensively. Erk can't even be detected properly from samples with a fixation time of 24 h using EXB but we obtained quite good results for all four samples if EXB Plus was used. Regarding β-actin it is eye-catching that we can't see any reduction of this protein using EXB Plus, however we do detect lower β-actin levels in samples from extensively fixed tissues extracted with EXB. These data clearly show that proteins can be efficiently recovered even after long-time fixation when using EXB Plus buffer.

### Protein extraction from long-term stored FFPE tissue blocks

**Figure 6:**
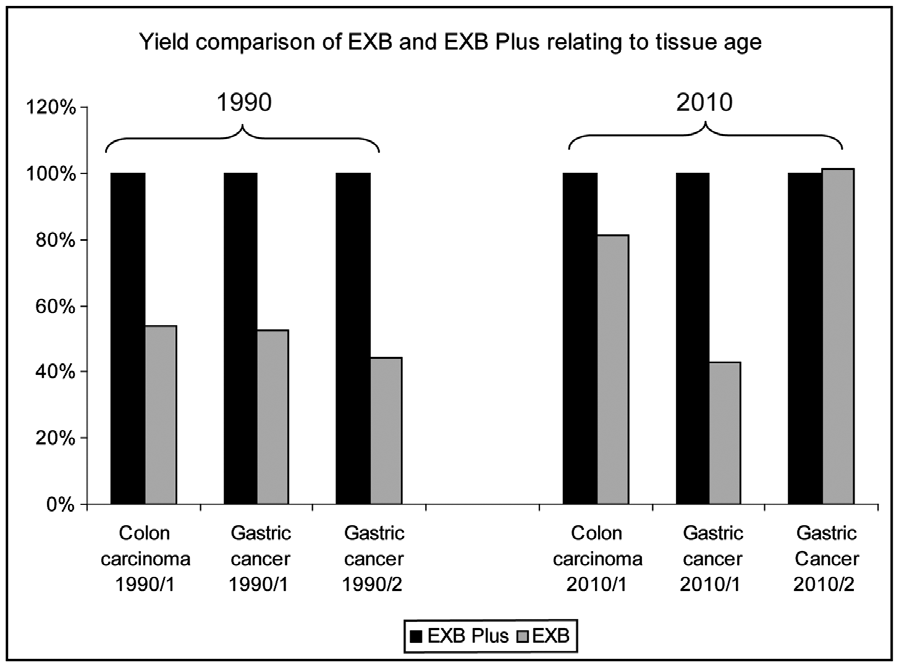
Protein extraction from FFPE samples from 1990 in comparison to samples from 2010. For both years the usage of EXB Plus resulted in higher protein yields compared to EXB but for the twenty years old samples the difference was more striking.

Another factor negatively affecting the protein amounts that can be recovered from FFPE tissue is the storage time of the tissue blocks. Formalin-fixed tissues may be stored over decades, with hardly any harm to the samples when inspected by histology. However, if proteins shall be extracted from long-term stored tissue blocks the protein yield decreases in comparison to short-term stored ones. This effect increases if the blocks have been cut before storage, which is the case for most FFPE blocks from clinical routine as they were used for diagnosis. Due to this reason our next attempt was to test the ability to extract proteins from such long-term stored tissues using EXB Plus. For this purpose we extracted one pair of colon carcinoma and two pairs of gastric cancer tissues from 1990 and 2010, respectively, with both EXB and EXB Plus. As shown in **Table 1** the protein amounts that could be extracted from the twenty years old blocks were lower (mean 1.26 mg/ml) compared to the yields from blocks from the year 2010 (mean 2.49 mg/ml) independently of the buffer used. However, if the two buffers are compared to each other one can see that protein yields from blocks from 2010 gained with EXB Plus are higher with a mean of 25% compared to blocks from the same year extracted with EXB (**Figure 6**). A comparison of the extractions from the blocks from 1990 revealed an even higher mean difference of 50% between the two buffers. These results show that especially for long-term stored tissues, much more protein can be recovered when using EXB Plus as extraction buffer instead of EXB.

**Table 1:** Protein yield from long-term stored FFPE tissue blocks.

Cases from 1990		Conc. in mg/ml	Cases from 2010		Conc. in mg/ml
Colon carcinoma	90/1 EXB	0.80	Colon carcinoma	10/1 EXB	2.24
Gastric cancer	90/1 EXB	0.98	Gastric cancer	10/1 EXB	1.39
Gastric cancer	90/2 EXB	0.75	Gastric cancer	10/2 EXB	2.67
**Mean**		**0.84**	**Mean**		**2.10**
Colon carcinoma	90/1 EXB Plus	1.48	Colon carcinoma	10/1 EXB Plus	2.76
Gastric cancer	90/1 EXB Plus	1.87	Gastric cancer	10/1 EXB Plus	3.24
Gastric cancer	90/2 EXB Plus	1.69	Gastric cancer	10/2 EXB Plus	2.63
**Mean**		**1.68**	**Mean**		**2.88**

Protein yields from one pair of colon carcinoma and two pairs of gastric cancer tissues from 1990 and 2010 were analysed. The same block was extracted twice, once with EXB and once with EXB Plus. The protein amounts from the newer blocks were higher than from the older ones. But for both time periods the amount extracted with EXB Plus was higher.

## Discussion

From the field of RNA extraction from FFPE tissues it is known that over-fixation of FFPE tissues can become an issue for extraction, particularly if fixation proceeds for longer than 24 hours, resulting in more irreversible crosslinking [15]–[17]. This could result in increased RNA degradation [18]. For the extraction of proteins this fact is also known, but notably less examined. Here we could show that using the new EXB Plus buffer, we are able to get reasonable amounts of proteins even from tissues that were fixed for 144 h. This is of great importance, as fixation times of routinely processed samples may vary from tissue sample to tissue sample. Now with EXB Plus all those samples, even the long-term fixed ones, could be made accessible for protein extraction. However, the known fact that the longer the tissue was fixed, the less protein can be extracted still applies.

Similar results were obtained for long-term stored tissues. Storage time is another factor that should be considered as a negative effector on extraction yields of macromolecules, like DNA, RNA or for us most important proteins that can be gained from FFPE tissue samples. These data are missing for alternative formalin-free fixatives. Even though it could be shown that storage time doesn't have an influence on RNA extraction [19], here we demonstrated that there is an influence on protein yield. We obtained about twice less proteins from samples stored since 1990 compared to the ones from the year 2010. But most interestingly we could show that using EXB Plus we get about 50% more proteins from samples from 1990 compared to EXB. For samples from the year 2010, EXB Plus got the higher yields too, but just with an advance of about 25%. Based on these results together with the fact that with EXB Plus we could also obtain higher protein amounts from long-term fixed samples, this new buffer system will be a very valuable tool for protein analysis of archival tissues. Additionally, we could show that EXB Plus is not only an improvement for such delicate FFPE blocks, but also for “normal”, routinely processed samples. Most importantly, all proteins analysed could be proven to be non-degraded, full-length, and immunoreactive.

Another important aspect of this study was to evaluate the suitability of EXB Plus for comparison of FFPE tissue to fresh frozen material. As the extraction from fresh frozen tissue is still the gold standard for protein analysis, it is often necessary to compare proteins from FFPE tissue to those extracted from cryo material. To really obtain similar protein compositions it is best to use the same buffer for both extractions. Here we could show that EXB Plus is also applicable to extract proteins from fresh frozen tissues and that the protein amount obtained with EXB Plus is as high as with standard extraction buffers especially designed for cryo material.

In conclusion, EXB Plus is the buffer of choice for protein extraction from FFPE tissues, particularly for demanding samples, such as long-term stored or extendedly fixed tissues. Tissue-based diagnostic testing is the gold standard for cancer diagnosis and is more and more dependent on increasing process standardization in the anatomic pathology laboratory and on improving laboratory workflows. Precise quantification of diagnostic or therapeutic proteins in FFPE cancer tissues is currently the greatest challenge for personalized cancer therapy. Protein lysates from FFPE tissue samples obtained with EXB Plus in combination with nano-scale quantitative downstream applications, such as reverse phase protein arrays, may help to solve current problems in protein biomarker quantification for cancer research, diagnosis, and treatment.

## Materials and Methods

### Ethic statement

All patients gave informed written consent and the study was approved by the Ethics Committee of the Technische Universität München, Munich, Germany.

### Tissue samples

We used one sample of each of the following human tissues, which had been routinely processed (formalin fixation in 4% neutral buffered formalin) in the Klinikum rechts der Isar of the Technische Universität München, Munich, Germany, from the years 1997 to 2007: Barrett's carcinoma, pancreas carcinoma, non-tumourous colon, gastric cancer and a lymph node sample. Additionally we used three different mamma carcinomas routinely processed of the Klinikum rechts der Isar and the Klinikum Rosenheim, respectively. For the extraction from mouse tissue female NMRI mice were maintained at the Charles River Laboratories in accordance with national and institutional guidelines for animal care. Each mouse was injected subcutaneously with each 200 µl of A431 (epidermoid carcinoma) or H1975 (non-small cell lung cancer) cell suspension (5×106 cells). When tumors reached a size of ∼1.5 cm mice were sacrificed and tumors were excised. The tissue was fixed in 4% (v/v) neutral buffered formalin for 24 h, rinsed in water for 2 h followed by paraffin embedding. Furthermore we analysed four lymph node samples which had been formalin fixed for 6 h, 24 h, 48 h or 144 h. For the comparison of FFPE tissue with fresh frozen tissue we used one non-tumourous colon tissue, one muscle sample, one non-tumourous stomach tissue and one ovarian carcinoma tissue. Each sample was divided into two equal parts, one of which was routinely formalin-fixed while the other one was snap-frozen in liquid nitrogen. To compare long-term stored tissues to recently fixed ones we used one routinely processed pair of colon carcinoma tissues and two pairs of routinely processed gastric cancer samples from the year 1990 and 2010, respectively. Reference haematoxylin/eosin stained sections of the tissues were histologically verified and areas of interest were marked by an experienced pathologist. Subsequent unstained sections of the same paraffin blocks were used for protein extraction.

### Protein extraction from FFPE tissues

We compared a new protein extraction buffer system (EXB Plus, Qiagen, Hilden, Germany) with an established extraction procedure (EXB, Qiagen, Hilden, Germany). Proteins were extracted according to the manufacturer's instructions. Briefly, after standard deparaffination of the tissue sections, the microdissected tissue of interest (as indicated in the haematoxylin/eosin stained reference sections) was transferred into EXB Plus buffer. We selected samples with the highest possible similarity regarding tissue area, tissue type, cell number, absence of necrosis and other factors for one to one comparison. After extraction according to the protocol the proteins were stored frozen at −20°C. To compare the new EXB Plus buffer against the established buffer system EXB, all extractions were also performed in this buffer as described before [5]. Comparative protein extractions from fresh frozen tissues were performed with both buffers (EXB Plus and EXB) and with another commercial buffer for protein extractions for fresh frozen tissue (T-Per, Thermo-Fisher, Rockford, USA). For all extractions applied: Approximately 1.5 mm^3^ tissue was processed in 100 µl of buffer (for FFPE-tissue 10 µm thick sections were used, for fresh frozen tissues we cut 20 µm sections). Protein concentrations were determined using the Bradford protein assay according to the manufacturer's instructions (BioRad, Hercules, USA). To calculate the protein yield in µg/mm^3^ we measured the sample area using the public domain software ImageJ (National Institutes of Health, USA).

### Western blot

Protein extracts were used for western blot analysis as previously described [20]. The tenth part of each extraction was applied to a 10% non-gradient SDS-gel. This gave us the possibility to compare the amount of individual proteins in the different extracts. Immunoblots were visualized with ECLplus (Amersham/GE Healthcare Europe GmbH, Munich, Germany).

### Antibodies

We studied the cell adhesion molecule E-Cadherin (#610182; BD Biosciences Pharmingen, San Diego, USA; 1∶5000), β-actin (A1978; Sigma, Hamburg, Germany; 1∶10000), the extracellular signal-regulated kinase Erk1/2 (#9102; Cell signalling, Danvers, USA; 1∶1000), the protein kinase B/Akt (#9272; Cell signalling, Danvers, USA; 1∶1000) and the heat shock protein Hsp70 (ab17850; Abcam, Cambridge, UK; 1∶50).

## References

[1] Ahram M, Flaig MJ, Gillespie JW, Duray PH, Linehan WM (2003). Evaluation of ethanol-fixed, paraffin-embedded tissues for proteomic applications.. Proteomics.

[2] Espina V, Mehta AI, Winters ME, Calvert V, Wulfkuhle J (2003). Protein microarrays: molecular profiling technologies for clinical specimens.. Proteomics.

[3] Gillespie JW, Best CJ, Bichsel VE, Cole KA, Greenhut SF (2002). Evaluation of non-formalin tissue fixation for molecular profiling studies.. Am J Pathol.

[4] Addis MF, Tanca A, Pagnozzi D, Crobu S, Fanciulli G (2009). Generation of high-quality protein extracts from formalin-fixed, paraffin-embedded tissues.. Proteomics.

[5] Becker KF, Schott C, Hipp S, Metzger V, Porschewski P (2007). Quantitative protein analysis from formalin-fixed tissues: implications for translational clinical research and nanoscale molecular diagnosis.. J Pathol.

[6] Becker KF, Mack H, Schott C, Hipp S, Rappl A (2008). Extraction of phosphorylated proteins from formalin-fixed cancer cells and tissues.. TOPATJ.

[7] Becker KF, Schott C, Becker I, Höfler H (2008). Guided protein extraction from formalin-fixed tissues for quantitative multiplex analysis avoids detrimental effects of histological stains.. Proteomics Clin Appl.

[8] Chu WS, Liang Q, Liu J, Wei MQ, Winters M (2005). A nondestructive molecule extraction method allowing morphological and molecular analyses using a single tissue section.. Lab Invest.

[9] Chung J, Lee SJ, Kris Y, Braunschweig T, Traicoff JL (2008). A well-based reverse-phase protein array applicable to extracts from formalin-fixed paraffin-embedded tissue.. Proteomics Clin Appl.

[10] Ikeda K, Monden T, Kanoh T, Tsujie M, Izawa H (1998). Extraction and analysis of diagnostically useful proteins from formalin-fixed, paraffin-embedded tissue sections.. J Histochem Cytochem.

[11] Nirmalan NJ, Harnden P, Selby PJ, Banks RE (2009). Development and validation of a novel protein extraction methodology for quantitation of protein expression in formalin-fixed paraffin-embedded tissues using western blotting.. J Pathol.

[12] Shi SR, Liu C, Balgley BM, Lee C, Taylor CR (2006). Protein extraction from formalin-fixed, paraffin-embedded tissue sections: quality evaluation by mass spectrometry.. J Histochem Cytochem.

[13] Chetty R, Serra S (2009). Loss of expression of E-cadherin in solid pseudopapillary tumors of the pancreas.. Pancreas.

[14] Pryczynicz A, Guzinska-Ustymowicz K, Kemona A, Czyzewska J (2010). Expression of the E-cadherin-catenin complex in patients with pancreatic ductal adenocarcinoma.. Folia Histochem Cytobiol.

[15] Bhudevi B, Weinstock D (2003). Detection of bovine viral diarrhea virus in formalin fixed paraffin embedded tissue sections by real time RT-PCR (Taqman).. J Virol Methods.

[16] Bresters D, Schipper ME, Reesink HW, Boeser-Nunnink BD, Cuypers HT (1994). The duration of fixation influences the yield of HCV cDNA-PCR products from formalin-fixed, paraffin-embedded liver tissue.. J Virol Methods.

[17] Macabeo-Ong M, Ginzinger DG, Dekker N, McMillan A, Regezi JA (2002). Effect of duration of fixation on quantitative reverse transcription polymerase chain reaction analyses.. Mod Pathol.

[18] Krafft AE, Duncan BW, Bijwaard KE, Taubenberger JK, Lichy JH (1997). Optimization of the Isolation and Amplification of RNA From Formalin-fixed, Paraffin-embedded Tissue: The Armed Forces Institute of Pathology Experience and Literature Review.. Mol Diagn.

[19] Specht K, Richter T, Muller U, Walch A, Werner M (2001). Quantitative gene expression analysis in microdissected archival formalin-fixed and paraffin-embedded tumor tissue.. Am J Pathol.

[20] Handschuh G, Candidus S, Luber B, Reich U, Schott C (1999). Tumour-associated E-cadherin mutations alter cellular morphology, decrease cellular adhesion and increase cellular motility.. Oncogene.

