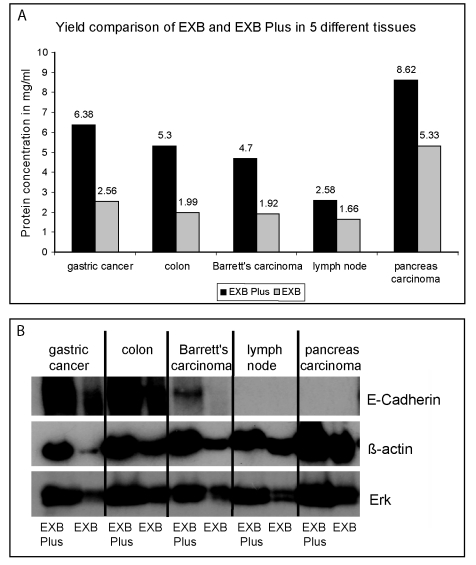# Correction: Successful Protein Extraction from Over-Fixed and Long-Term Stored Formalin-Fixed Tissues

**DOI:** 10.1371/annotation/a42e114f-a708-4423-8a3e-a1d8919b9b60

**Published:** 2011-04-29

**Authors:** Claudia Wolff, Christina Schott, Peter Porschewski, Bilge Reischauer, Karl-Friedrich Becker

The published version of Figure 1 is incorrect. View the correct Figure 1 here: 

**Figure:**